# LRRC8 channel complexes counterbalance K_ATP_ channels to mediate swell-secretion coupling in mouse pancreatic **β** cells

**DOI:** 10.1172/jci.insight.188020

**Published:** 2025-04-29

**Authors:** Tarek Mohamed Abd El-Aziz, Chen Kang, Litao Xie, John D. Tranter, Sumit Patel, Rahul Chadda, Maria S. Remedi, Rajan Sah

**Affiliations:** 1 Department of Internal Medicine, Cardiovascular Division, Washington University School of Medicine, St. Louis, Missouri, USA.; 2 Zoology Department, Faculty of Science, Minia University, El-Minia, Egypt.; 3 Department of Medicine, Division of Endocrinology, Metabolism and Lipid Research, Washington University School of Medicine, St. Louis, Missouri, USA.; 4 St. Louis VA Medical Center, St. Louis, Missouri, USA.

**Keywords:** Cell biology, Metabolism, Beta cells, Insulin, Ion channels

## Abstract

Insulin secretion from pancreatic β cells is initiated by membrane potential depolarization, followed by activation of voltage-gated Ca^2+^ channels to trigger Ca^2+^-mediated insulin vesicle fusion with the β cell plasma membrane. Here, we show that β cell swelling associated with glucose metabolism was sensed by LRRC8 channel complexes and contributed to insulin secretion. Hypertonic perfusate (360–380 mOsm) dose dependently impaired glucose-stimulated insulin secretion by counteracting β cell swelling. Hypotonic perfusate alone, independent of glucose stimulation or K_ATP_ channel closure, was sufficient to increase β cell intracellular Ca^2+^ and trigger insulin secretion. Inhibition of sodium-potassium-chloride cotransporter-1 with bumetanide, which diminished the intracellular Cl^–^ concentration in β cells and consequently reduced Cl^–^ efflux via LRRC8 channel complexes, also significantly reduced hypotonic-stimulated insulin secretion. Finally, stimulation of insulin secretion by the glucokinase activator GKA50, which is known to induce β cell swelling, was entirely suppressed in β cell–targeted *Lrrc8a* KO islets. These data support a model wherein the LRRC8 channel complex senses β cell swelling triggered by glucose metabolism and regulates β cell insulin secretion through activation of LRRC8-mediated Cl^–^ efflux.

## Abstract 2

LRRC8 channels sense β-cell swelling in response to glucose metabolism to activate a depolarizing Cl- conductance which counterbalances hyperpolarizing KATP to regulate insulin secretion

## Introduction

Insulin secretion from pancreatic β cells is essential for maintaining blood glucose homeostasis. In glucose-stimulated insulin secretion (GSIS), as blood glucose levels increase, glucose enters pancreatic β cells via glucose transporters, followed by glucokinase-mediated phosphorylation and conversion to pyruvate via glycolysis (1, 2). Glycolysis and subsequent oxidative metabolism generate ATP within the cell, leading to an increased ATP/ADP ratio, which results in the closure of ATP-sensitive K^+^ (K_ATP_) channels and membrane depolarization (3, 4). Finally, membrane depolarization induces Ca^2+^ influx via activation of voltage-gated Ca^2+^ channels (VGCCs), resulting in exocytosis of insulin-containing granules (5, 6), which promotes glucose uptake from the circulation by insulin-sensitive tissues (liver, skeletal muscle, adipose). While it is widely accepted that closure of K_ATP_ channels due to an increased ATP/ADP ratio is required for GSIS, other mechanisms, independent of K_ATP_ channel closure activity, have also been proposed to contribute to GSIS. Since the mid-1990s, a growing body of evidence suggested that activation of anionic currents may contribute to glucose-induced depolarization of β cells, independent of K_ATP_ channel activity (7–19). Collectively these studies hypothesized that glucose metabolism increases intracellular osmolarity and cell volume, leading to subsequent activation of a volume-sensitive, outwardly rectifying chloride conductance mediated by the volume-regulated anion channel (VRAC), which contributes to β cell depolarization required for insulin secretion (18). While VRAC-mediated chloride currents were identified in 1988 as a mechanism for regulatory volume decrease (20), it was not until 2014 that two independent studies discovered the molecular identity of VRAC (21, 22). It is now known that VRAC is a heterohexameric channel complex formed by at least one LRRC8A (also known as SWELL1) essential subunit in combination with LRRC8B, -C, -D, and/or -E subunits. Since the molecular identity of VRAC was elucidated, studies by us and others have demonstrated the importance of SWELL1/VRAC in β cell excitability and insulin secretion (23–25).

The aim of this study was to further explore the mechanism of “swell-secretion” coupling that is mediated by LRRC8 channel complexes in β cells. Here, we show that attenuation of cell swelling and therefore LRRC8 activation by extracellular hypertonic conditions or through inhibition of water influx via aquaporin-7 impaired GSIS and that an adequate intracellular chloride concentration to allow for Cl^–^ efflux–mediated β cell depolarization by LRRC8 channels was required for both glucose-stimulated and hypotonic-simulated insulin secretion. Furthermore, we show that the activation of LRRC8 channels was required for glucokinase-mediated potentiation of insulin secretion and that LRRC8 activation by hypotonic swelling was sufficient to induce calcium influx and insulin secretion independent of K_ATP_ closure. Overall, these results further implicate LRRC8 channel complexes as sensors of β cell swelling triggered by glucose metabolism, which regulates insulin secretion through activation of LRRC8-mediated chloride efflux.

## Results

### Hypertonicity suppresses glucose-induced insulin secretion.

**Figure 1:**
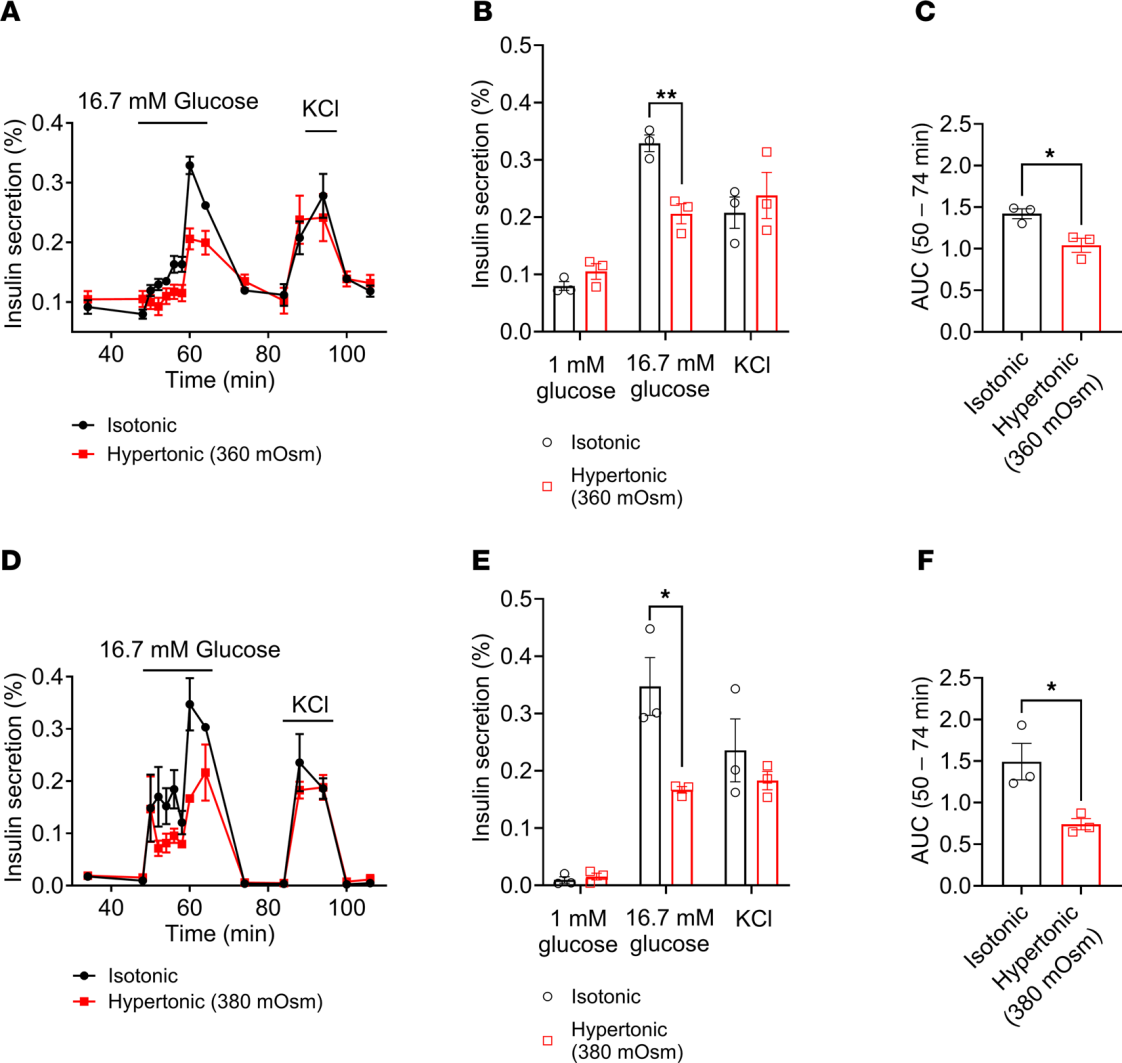
Counteracting glucose-induced β cell swelling with increasingly hypertonic perfusate (360–380 mOsm) dose dependently impairs glucose-stimulated insulin secretion. Glucose-stimulated insulin secretion was induced by perfusing 50 islets isolated from WT mice (*n* = 3–5) with 16.7 mM glucose under isotonic (300 mOsm) and hypertonic (**A**, 360 mOsm; **D**, 380 mOsm) conditions. Insulin secretion is represented as a percentage of total insulin content. (**B** and **E**) Mean (%) insulin secretion in response to 1 mM glucose (40 min), 16.7 mM glucose (60 min), and 40 mM KCl (88 min), under isotonic and hypertonic conditions. (**C** and **F**) Mean AUC for the percentage of insulin secretion during both first-phase and second-phase (50–74 min) responses to high glucose (16.7 mM) under isotonic and hypertonic conditions. Data are represented as mean ± SEM. Statistical significance for all data was determined using 2-tailed unpaired Student’s *t* test (*P < 0.05; **P < 0.01).

Our previous study demonstrated that LRRC8 channels mediate a glucose-stimulated depolarizing chloride conductance that contributes to β cell depolarization, activation of voltage-gated calcium channels (VGCCs), and subsequent insulin secretion via Ca^2+^-dependent insulin vesicle fusion (23). Importantly, this model proposes that LRRC8 channel activation is triggered by β cell swelling due to the increase in intracellular osmolarity associated with glucose metabolism. However, this swelling may be offset by elevated extracellular osmolarity, despite elevated concentrations of intracellular glucose metabolites. Therefore, we asked if swelling itself is an essential component of GSIS. To test this, we investigated the effects of extracellular hypertonicity on insulin secretion in isolated mouse islets (Figure 1). Exposure of islets to standard stimulatory glucose (16.7 mM) resulted in both first- and late-phase insulin secretion, which rapidly returned to basal levels when stimulatory glucose perfusate was returned to 1 mM (Figure 1, A and D). We found that insulin secretion was dose dependently suppressed by inhibiting β cell swelling using hypertonic solutions of increasing osmolarity. Under these conditions, 360 mOsm/kg hypertonic solution reduced peak GSIS from 0.33% ± 0.02% to 0.21% ± 0.02% (Figure 1, A and B), and insulin release, as assessed by the cumulative AUC during the 50- to 74-minute period of the perifusion assay, was reduced by 37.5% (Figure 1, A–C). While 380 mOsm/kg hypertonic solution reduced peak GSIS from 0.35% ± 0.05% to 0.17% ± 0.006% (Figure 1, D and E) and reduced insulin release, as assessed by the cumulative AUC during the 50- to 74-minute period of the perifusion assay, by 52% (Figure 1, D–F). These data reveal the contribution of cell swelling to GSIS in pancreatic β cells.

### Sufficiently elevated intracellular Cl^–^ is required for both glucose- and hypotonic-stimulated insulin secretion in β cells.

In our previous study, we reported that LRRC8 channels mediate a glucose- and swell-activated depolarizing chloride current (I_Cl,SWELL_) that contributes to β cell depolarization due to the higher intracellular chloride concentration (~34 mM) of pancreatic β cells (23). This results in a reversal potential for chloride (~–30 mV) that is well above the β cell resting membrane potential (–70 mV) and near the activation threshold potential for L-type Ca^2+^ channels. Efflux of Cl^–^ from β cells therefore induces membrane depolarization, which contributes to activation of L-type Ca^2+^ channels and subsequent insulin vesicle fusion with the plasma membrane.

**Figure 2:**
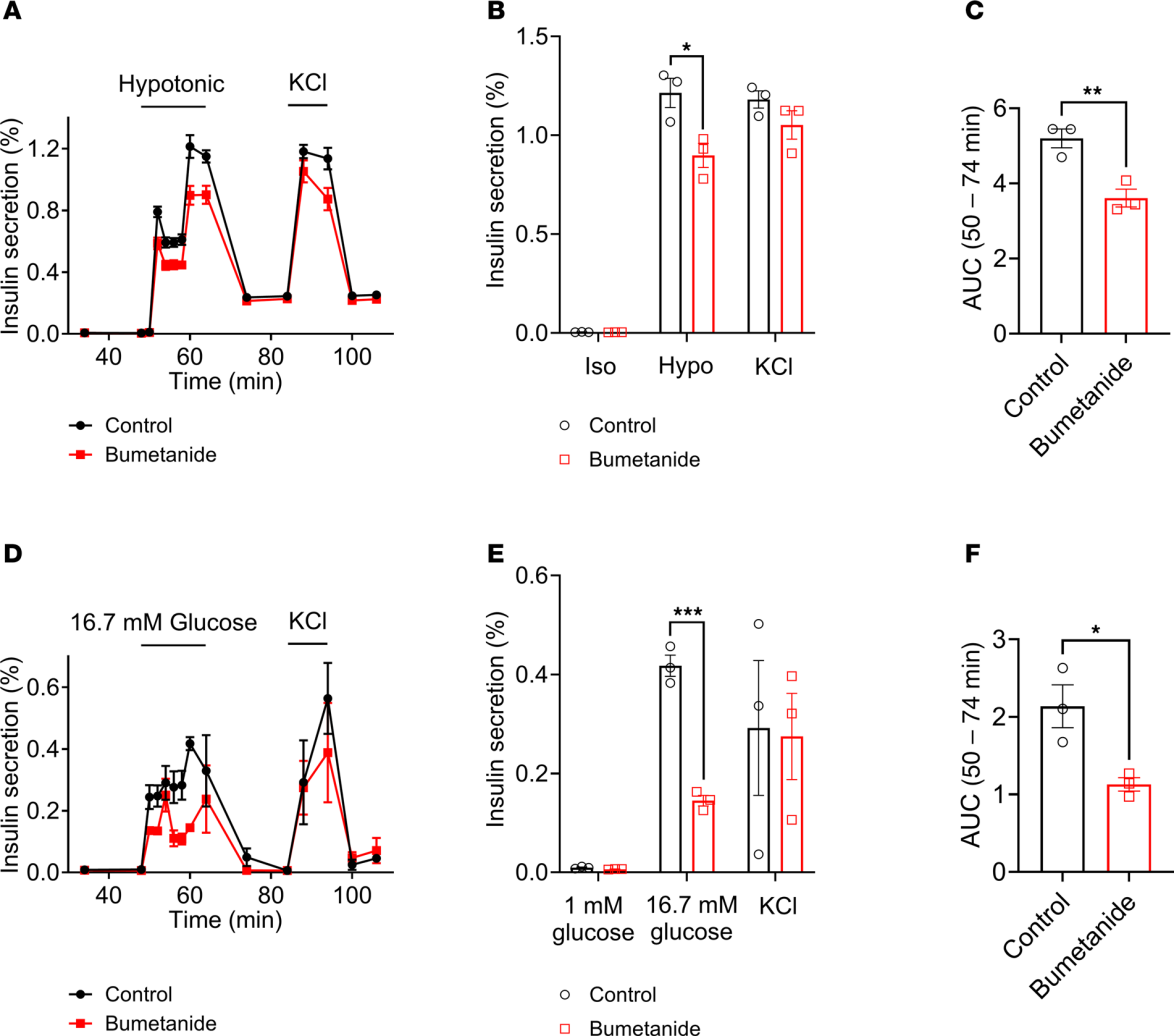
Elevated β cell intracellular chloride augments hypotonic- and glucose-stimulated insulin secretion. (**A**) Hypotonicity-stimulated insulin secretion was performed by perifusion of 50 islets from WT mice (*n* = 3) under control conditions (0 mM glucose, 210 mOsm) or in the presence of the NKCC1 inhibitor bumetanide (10 μM). Insulin secretion is represented as a percentage of total insulin content. (**B**) Mean (%) insulin secretion in response to isotonic solution (48 min), hypotonic (210 mOsm) solution (60 min), and 40 mM KCl (88 min) in the absence or presence of bumetanide. (**C**) Mean AUC for the percentage of insulin secretion during both first-phase and second-phase (50–74 min) responses to hypotonic solution (210 mOsm). (**D**) Glucose-stimulated insulin secretion (16.7 mM glucose) in the absence and presence of bumetanide. (**E**) Mean (%) insulin secretion in response to 1 mM glucose (48 min), 16.7 mM glucose (60 min), and 40 mM KCl (88 min) in the absence or presence of bumetanide. (**F**) Mean AUC for the percentage of insulin secretion during both first-phase and second-phase (50–74 min) responses to glucose stimulation (16.7 mM) in the absence or presence of bumetanide. Data are represented as mean ± SEM. Statistical significance for all data was determined using 2-tailed unpaired Student’s *t* test (*P < 0.05; **P < 0.01; ***P < 0.001).

To determine the contributions of elevated intracellular [Cl^–^] to LRRC8-mediated Cl^–^ efflux and insulin secretion, we measured both hypotonic swelling-induced insulin secretion (Figure 2A) and GSIS (Figure 2D) in the presence and absence of bumetanide, an inhibitor of Na^+^-K^+^-2Cl^–^ cotransporter isoform 1 (NKCC1), which maintains intracellular chloride in murine β cells (16, 26, 27). Bumetanide treatment led to a significant reduction in peak hypotonic swelling–induced insulin secretion from 1.21% ± 0.07% to 0.89% ± 0.06% (Figure 2, A and B). Insulin release, as assessed by the cumulative AUC during the 50- to 74-minute period of the perifusion assay, was reduced by 30.5% with bumetanide treatment following hypotonic stimulation (Figure 2C). Similarly, bumetanide significantly reduced peak GSIS from 0.42% ± 0.02% to 0.14% ± 0.01% (Figure 2, D and E). Insulin release, as measured by cumulative AUC, was reduced by 47.2% with bumetanide treatment following high-glucose stimulation (Figure 2F). Although previous studies have demonstrated that the inhibitory effects of bumetanide on β cell GSIS are mediated by a reduction in β cell intracellular Cl^–^ (16), as opposed to an off-target effect, we sought to confirm that bumetanide in fact reduced intracellular Cl^–^ in β cells. To measure intracellular Cl^–^ concentrations in β cells, we used MQAE, a chloride-sensitive fluorescent dye that reports intracellular Cl^–^ concentration through its collisional quenching properties, whereby MQAE fluorescence quenches in response to increasing intracellular Cl^–^ concentrations. This approach has been widely applied to study intracellular Cl^–^ dynamics in various cell types, including olfactory neurons, dorsal root ganglion cells, salivary glands, and brain slices (28–31). MQAE-based intracellular Cl^–^ measurements reveal significantly higher MQAE fluorescence intensity in bumetanide-treated β cells (1,367.2 ± 116.3 AU, *n* = 20) as compared with control β cells (1,007.9 ± 49.5 AU, *n* = 20; Supplemental Figure 1; supplemental material available online with this article; https://doi.org/10.1172/jci.insight.188020DS1), suggesting that bumetanide treatment significantly reduces intracellular Cl^–^ concentration in β cells. We next measured normalized MQAE fluorescence in control versus bumetanide-treated cells upon perfusion of a low-Cl^–^ solution (24 mM) to promote Cl^–^ efflux (Supplemental Figure 2, A–C). As control β cells with active NKCC1 transporters have an intracellular [Cl^–^] of approximately 34 mM (32) under standard extracellular Cl^–^ concentrations of approximately 140 mM, reducing the extracellular Cl^–^ to 24 mM should result in a net efflux of Cl^–^ and therefore an increase MQAE fluorescence. Indeed, we observed a larger increase in MQAE fluorescence signal (Supplemental Figure 2, A–C) and a greater rate of change of MQAE fluorescence (Supplemental Figure 2, A–D) in control cells as compared with bumetanide-treated cells. This is consistent with lower intracellular Cl^–^ concentrations in the bumetanide-treated cells, due to NKCC inhibition reducing Cl^–^ accumulation and limiting Cl^–^ efflux when extracellular Cl^–^ is reduced to 24 mM. Next, we repeated this experiment, measuring normalized fluorescence intensity while transitioning β cells from media (~140 mM Cl^–^) to a low-extracellular Cl^–^ solution (24 mM) and then back to a higher-extracellular Cl^–^ solution (135 mM). Similar to that shown in Supplemental Figure 2, A–C, our results show a larger and more rapid increase in normalized fluorescence intensity in control cells as compared with bumetanide-treated cells during perfusion with the low-Cl^–^ solution. Conversely, there was more pronounced quenching of MQAE fluorescence in the bumetanide-treated β cells as compared with control cells upon switching to the high-Cl^–^ extracellular solution (Supplemental Figure 2, E–G). These data are consistent with a reduction in intracellular Cl^–^ in the bumetanide-pretreated β cells from the control value of approximately 34 mM to 24 mM, thereby reducing the driving force for Cl^–^ efflux upon application of 24 mM extracellular Cl^–^ and increasing the driving force for Cl^–^ influx upon application of 135 mM extracellular Cl^–^. Taken together, these results support the model that elevated intracellular chloride maintained via NKCC1 activity contributes to both hypotonic swelling- and glucose-induced insulin secretion in murine islets.

### Water influx via β cell aquaporin-7 contributes to glucose-induced insulin secretion.

**Figure 3:**
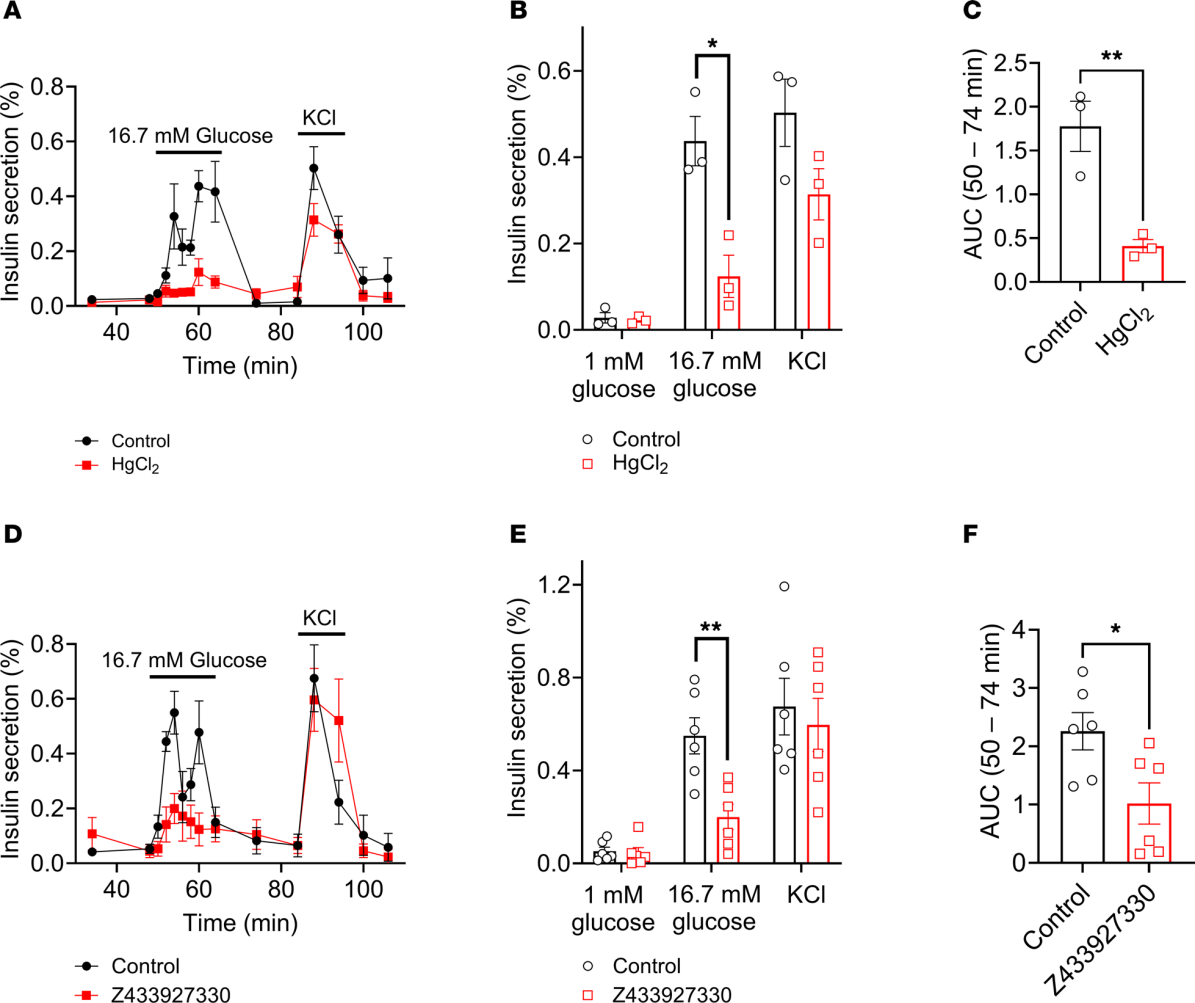
Pancreatic β cell aquaporin channel activity contributes to glucose-stimulated insulin secretion. (**A** and **D**) Glucose-stimulated insulin secretion was performed by perifusion of 50 islets from WT mice (*n* = 3) under control conditions (16.7 mM glucose, 300 mOsm) or in the presence of 0.3 mM HgCl_2_ (**A**) or 200 nM Z433927330 (**D**) (16.7 mM glucose, 300 mOsm). Insulin secretion is represented as a percentage of total insulin content. (**B** and **E**) Mean (%) insulin secretion in response to 1 mM glucose (48 min), 16.7 mM glucose (60 min), and 40 mM KCl (88 min) in the absence or presence of 0.3 mM HgCl_2_ (**B**) or 200 nM Z433927330 (**E**). (**C** and **F**) Mean AUC for the percentage of insulin secretion during the first-phase and second-phase (50–74 min) responses to glucose stimulation (16.7 mM) in the absence or presence of 0.3 mM HgCl_2_ (**C**) or 200 nM Z433927330 (**F**). Data are represented as mean ± SEM. Statistical significance for all data was determined using 2-tailed unpaired Student’s *t* test (*P < 0.05; **P < 0.01).

Aquaporins are a family of 13 channels (AQP0–12) that facilitate the rapid transport of water across cell membranes. AQP7, an aquaglyceroporin that permeates both water and glycerol, is expressed in pancreatic β cells (33) and has been shown to contribute to β cell insulin secretion (34–36). We hypothesized that increased intracellular β cell osmolarity associated with glucose metabolism draws free water through AQP7 down the osmotic gradient, contributing to β cell swelling and LRRC8-mediated insulin secretion. To investigate if water influx via AQP7 contributes to LRRC8-mediated swelling-secretion coupling in pancreatic β cells, we applied mercury chloride (HgCl_2_), a general AQP inhibitor, and Z433927330, a selective AQP7 inhibitor, to isolated murine islets to block AQP7 during GSIS. AQP inhibition with HgCl_2_ markedly reduced both phase 1 and 2 insulin secretion (Figure 3A), decreasing GSIS from 0.44% ± 0.06% to 0.12% ± 0.05% (Figure 3B). Insulin secretion, as assessed by cumulative AUC, was reduced by 77% with HgCl_2_ treatment in response to high-glucose stimulation (Figure 3C). Similarly, selective AQP7 inhibition using Z433927330 significantly reduced both phase 1 and phase 2 insulin secretion (Figure 3D), decreasing GSIS from 0.55% ± 0.08% to 0.17% ± 0.07% (Figure 3E). Insulin secretion, as assessed by cumulative AUC, was reduced by 56.5% following Z433927330 treatment in response to high-glucose stimulation (Figure 3F). These data demonstrate that AQP7-mediated water influx contributes to LRRC8-mediated swell-secretion coupling in pancreatic β cells.

### LRRC8 channels are required for GKA50-mediated potentiation of insulin secretion.

GKA50, a glucokinase activator, potentiates insulin secretion by increasing the rate of glucose metabolism within β cells, thus increasing the intracellular ATP/ADP ratio with subsequent K_ATP_ channel closure, membrane depolarization, and L-type Ca^2+^ channel activation (37). However, GKA50 also increases the accumulation of glucose metabolites within β cells, which promotes osmotic swelling (19). We therefore hypothesized that GKA50-stimulated β cell swelling promotes insulin secretion via activation of LRRC8 channels. First, we measured the β cell cross-sectional area (indicative of cell volume) at substimulatory glucose concentrations (4 mM) in the presence or absence of GKA50 in both WT and LRRC8A-KO β cells. WT β cells were obtained from *Lrrc8a*^fl/fl^ (also known as *Swell1*^fl/fl^) islets transduced with an adenovirus expressing the genetically encoded Ca^2+^ indicator, GCaMP6s, under a rat insulin promoter (Ad-RIP1-GCaMP6s, referred to as WT) while LRRC8A-KO β cells were generated from *Lrrc8a*^fl/fl^ islets transduced with an adenovirus expressing both GCaMP6s and Cre recombinase (Ad-RIP1-Cre-P2A-GCaMP6s, referred to as LRRC8A KO).

**Figure 4:**
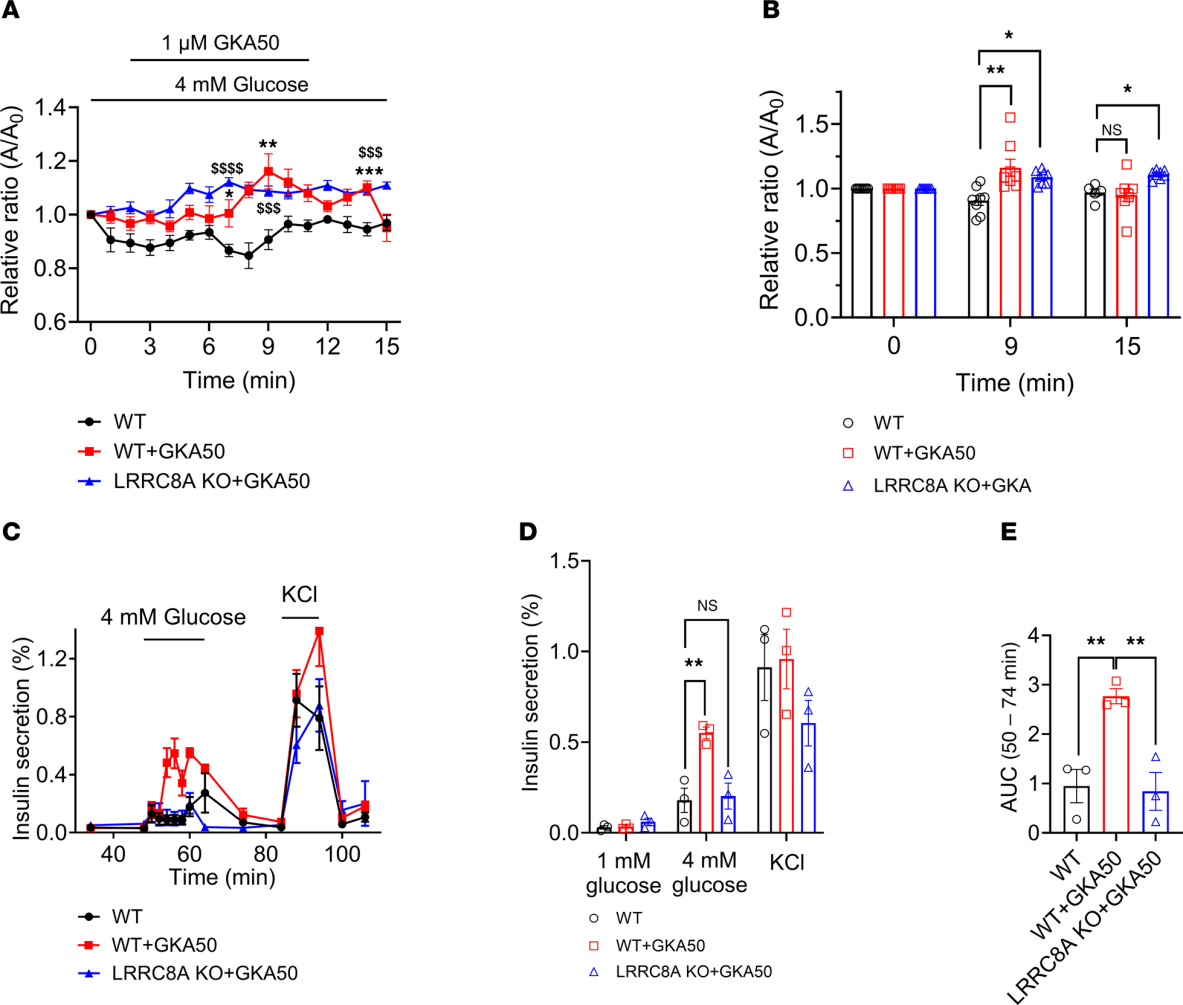
Glucokinase activator GKA50 induces β cell swelling and requires LRRC8A to potentiate insulin secretion. (**A**) Cross-sectional area of WT and LRRC8A-KO β cells in response to glucose (4 mM) simulation, in the presence of 1 μM glucokinase activator GKA50, as compared with untreated WT controls. (**B**) Mean cross-sectional area of WT and LRRC8A-KO β cells in the presence of 1 μM GKA50, as compared with untreated WT controls, at 0, 9, and 15 minutes of glucose (4 mM) stimulation. (**C**) Glucose-stimulated insulin secretion from 50 islets isolated from WT mice (*n* = 3–4) under control conditions (4 mM glucose, 300 mOsm); WT islets treated with 1 μM GKA50 (4 mM glucose, 300 mOsm) and islets isolated from LRRC8A-KO mice (*n* = 3–4) treated with 1 μM GKA50 (4 mM glucose, 300 mOsm). Insulin secretion is represented as a percentage of the total insulin content. (**D**) Mean (%) insulin secretion from WT and LRRC8A-KO islets treated with 1 μM GKA50, as compared with untreated WT controls, in response to 1 mM glucose (48 min), 4 mM glucose (60 min), and 40 mM KCl (88 min). (**E**) Mean AUC for the percentage of insulin secretion during the first-phase and second-phase (50–74 min) responses to 4 mM glucose in the absence or presence of 1 μM GKA50. Data are represented as mean ± SEM. Statistical significance was evaluated using either a 2-tailed unpaired Student’s *t* test (for normally distributed data) or the Mann-Whitney test (for nonparametric comparisons)) or 1-way ANOVA (when comparing 3 or more groups) (*^,$^*P* < 0.05; **P < 0.01; ***^,$$$^*P* < 0.001; ^$$$$^*P* < 0.0001). *WT vs. WT+GKA50; ^$^WT vs. LRRC8A KO+GKA50.

GKA50 application to WT β cells induced increases in β cell cross-sectional area, as compared with that of control (Figure 4, A and B). Notably, increases in cell size were sustained during GKA50 exposure and only returned to baseline upon removal of GKA50. Additionally, LRRC8A-KO β cells also exhibited GKA50-induced increases in cell size; however, these increases remained following removal of GKA50, which is consistent with impaired regulatory volume decrease due to loss of LRRC8A (22).

Next, to determine the contribution of LRRC8 channels to GKA50-mediated potentiation of insulin secretion, we assessed GSIS in the presence of glucose with or without GKA50, in isolated WT and LRRC8A-KO islets. Application of substimulatory (4 mM) glucose induced robust first- and late-phase insulin secretion in WT islets treated with GKA50 as compared with vehicle-treated controls (Figure 4, C and D). Interestingly, GKA50 failed to induce GSIS in the absence of LRRC8A with results comparable to WT islets without GKA50 stimulation (Figure 4, C and D). GKA50 led to a significant increase in glucose-induced insulin secretion in WT mice, increasing cumulative AUC from 0.95 ± 0.33 to 2.77 ± 0.15 (Figure 4E). These data reveal that LRRC8A is required for GKA50-mediated potentiation of GSIS likely by sensing GKA50-mediated β cell swelling.

### Hypotonic swelling induces β cell calcium transients independent of K_ATP_ closure.

**Figure 5:**
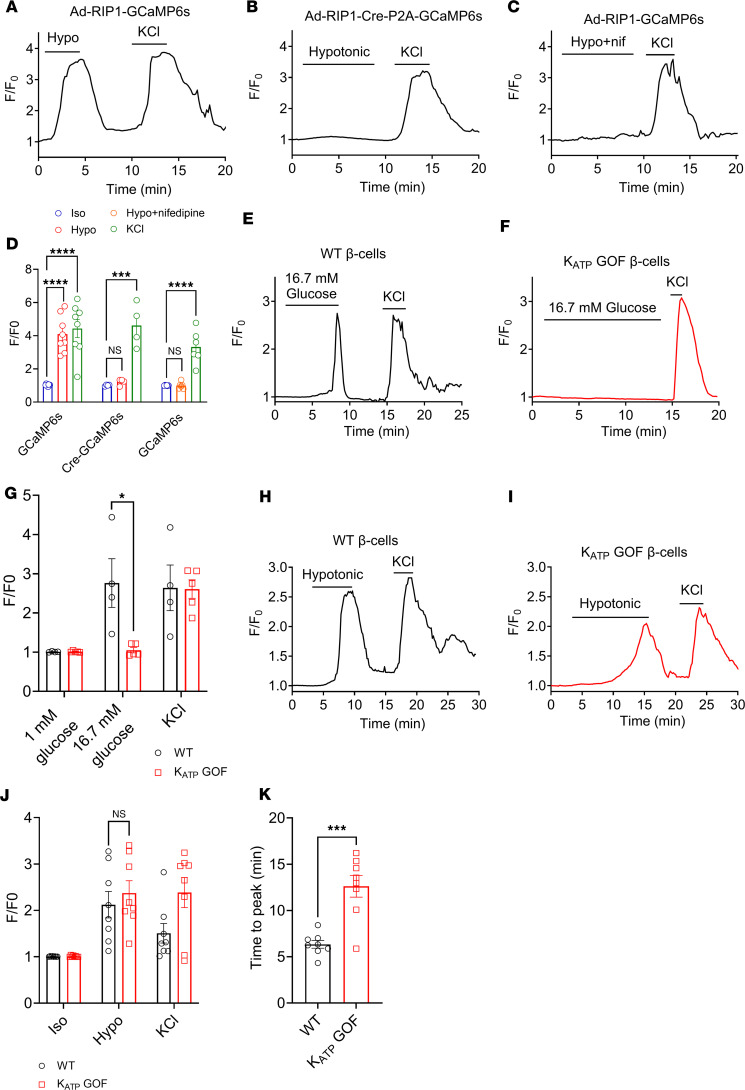
Hypotonic swelling-induced calcium transients in β cells occur independently of K_ATP_ channel closure. (**A**–**C**) Representative GCaMP6s Ca^2+^ transients in WT (**A**, Ad-RIP1-GCaMP6s/*Lrrc8a^fl/fl^*), LRRC8A-KO (**B**, Ad-RIP1-Cre-P2A-GCaMP6s/*Lrrc8a^fl/fl^*), and WT+nifedipine (**C**, 10 μM) primary murine β cells in response to hypotonic swelling stimulation (210 mOsm/kg; isotonic 300 mOsm/kg) and 40 mM KCl stimulation. (**D**) Mean relative intensity of GCaMP6s Ca^2+^ transients in WT (*n* = 8), LRRC8A-KO (*n* = 4), and WT+nifedipine (*n* = 6) β cells following hypotonic (210 mOsm) stimulation and KCl (40 mM) treatment. (**E** and **F**) Representative GCaMP6s Ca^2+^ transients in WT (**E**) and K_ATP_ GOF mutant (**F**) β cells in response to high (16.7 mM) glucose, under isotonic conditions. (**G**) Mean relative intensity of GCaMP6s Ca^2+^ transients in WT (*n* = 4) and K_ATP_ GOF mutant (*n* = 5) β cells following glucose stimulation (16.7 mM) and KCl (40 mM) treatment. (**H** and **I)** Representative GCaMP6s Ca^2+^ transients in WT (**H**) and K_ATP_ GOF mutant (**I**) β cells in response to hypotonic stimulation (210 mOsm, 0 mM glucose). (**J**) Mean relative intensity of GCaMP6s Ca^2+^ transients in WT (*n* = 8) and K_ATP_ GOF mutant (*n* = 8) β cells following hypotonic (210 mOsm) stimulation and KCl (40 mM) treatment. (**K**) Time-to-peak histogram showing delayed Ca^2+^ responses in K_ATP_ GOF β cells during hypotonic swelling as compared with WT controls. Data are represented as mean ± SEM. Statistical significance for all data was determined using 2-tailed unpaired Student’s *t* test or 1-way ANOVA (when comparing 3 or more groups) (*P < 0.05; ***P < 0.001; ****P < 0.0001).

Rising intracellular glucose and its metabolism induces β cell swelling, which both activates LRRC8-mediated depolarizing I_Cl,SWELL_ and increases the intracellular ATP/ADP ratio, leading to K_ATP_ channels closure, reduced hyperpolarizing I_KATP_, and consequent β cell depolarization followed by L-type Ca^2+^ channel activation and insulin vesicle fusion. To dissect the contribution of swell-mediated depolarization via I_Cl,SWELL_ versus β cell depolarization following K_ATP_ closure, we first studied how cell swelling influences Ca²^+^ signaling in β cells under hypotonic conditions (220 mOsm/kg) without glucose stimulation (0 mM). These conditions were chosen to specifically activate LRRC8-mediated I_Cl,SWELL_ while keeping K_ATP_ channels open, allowing us to separate the effects of I_Cl,SWELL_ activation from K_ATP_ channel closure. To measure LRRC8-related Ca²^+^ signaling in primary mouse β cells, we used adenoviruses to introduce a genetically encoded calcium sensor, GCaMP6s, under the control of the rat insulin promoter 1 (RIP1). This was done either alone (Ad-RIP1-GCaMP6s, referred to as WT) or alongside Cre-recombinase (Ad-RIP1-Cre-P2A-GCaMP6s, referred to as LRRC8A KO) to delete LRRC8A in β cells from *Lrrc8a*^fl/fl^ mice. This method ensured that the calcium sensor was expressed only in β cells while also allowing targeted deletion of LRRC8A. We found that exposing WT β cells (Ad-RIP1-GCaMP6s/*Lrrc8a*^fl/fl^) to hypotonic conditions triggered strong calcium signals, which quickly returned to normal when isotonic conditions were restored (Figure 5, A and D). However, β cells lacking LRRC8A (Ad-RIP1-Cre-P2A-GCaMP6s/*Lrrc8a*^fl/fl^) showed no calcium response to hypotonic swelling (Figure 5, B and D), even though their calcium response to KCl stimulation remained intact. This confirms that LRRC8A is responsible for generating the swell-activated depolarizing current in β cells. Furthermore, we found that inhibiting VGCCs with nifedipine completely prevented the hypotonic swelling-induced calcium response (Figure 5, C and D). This suggests that cell swelling leads to β cell membrane depolarization, which, in turn, activates VGCC-mediated calcium entry to trigger insulin vesicle fusion, rather than other mechanoresponsive calcium influx pathways such as TRP channels.

Additionally, we used islets from the K_ATP_ gain-of-function (GOF) murine model *Rosa26-Kir6.2[K185Q,ΔN30]* (38). Islets from *Rosa26-Kir6.2[K185Q,ΔN30]* transgenic mice were transduced with Ad-RIP1-GCaMP6s, WT, or an adenovirus expressing both GCaMP6s and Cre recombinase (Ad-RIP1-Cre-P2A-GCaMP6s, referred to as K_ATP_ GOF). Importantly, those expressing Cre recombinase express K_ATP_ channels that are insensitive to elevations in the intracellular ATP/ADP ratio and remain open even in the presence of elevated extracellular glucose levels (38), thus suppressing glucose-stimulated rises in β cell intracellular Ca^2+^ and subsequent insulin secretion. We first confirmed that glucose-stimulated Ca^2+^ transients elicited in control β cells with normal K_ATP_ function (WT, Figure 5, E and G) were abrogated in K_ATP_ GOF β cells (Figure 5, F and G), consistent with previous reports (3, 39). Next, we examined the capacity of β cell swelling and LRRC8 channel activation alone to stimulate intracellular Ca^2+^ transients in β cells without glucose-stimulated K_ATP_ closure by applying hypotonic solution without glucose to WT cells. Indeed, hypotonic swelling alone triggered robust Ca^2+^ transients in WT β cells in the absence of glucose-stimulated K_ATP_ channel closure (Figure 5, H and J), consistent with previous reports of a glucose-independent, swell-mediated mechanism of β cell insulin secretion (8, 40). To further test the ability of cell swelling and LRRC8-mediated depolarizing I_Cl,SWELL_ to overcome hyperpolarizing K_ATP_, we repeated this experiment using K_ATP_ GOF β cells. Hypotonic solution without glucose was sufficient to induce intracellular Ca^2+^ transients in K_ATP_ GOF β cells (Figure 5, I and J), indicating that β cell swelling and LRRC8-mediated depolarizing I_Cl,SWELL_ can overcome constitutive K_ATP_ activity. Interestingly, the time to peak of intracellular Ca^2+^ in response to hypotonic swelling in K_ATP_ GOF β cells was significantly longer than that in control cells with regular K_ATP_ channels (Figure 5K), consistent with a counterregulatory relationship between LRRC8-mediated depolarization and K_ATP_-mediated hyperpolarization. Constitutively open K_ATP_ channels in GOF β cells maintain a hyperpolarized membrane potential that prevents voltage-dependent Ca²^+^ channel (VDCC) activation (41). For Ca²^+^ transients to occur, LRRC8-mediated depolarizing currents need to overcome K_ATP_-mediated hyperpolarization and shift the membrane potential to the VDCC activation threshold. The delayed Ca²^+^ response in our experiments aligns well with the known time course of VRAC activation under hypotonic conditions, supporting the model that LRRC8-mediated currents are gradually overcoming the opposing K_ATP_ currents to drive β cell depolarization (24). Collectively, these data demonstrate that hypotonic swelling alone induces a sufficiently strong depolarizing stimulus via I_Cl,SWELL_ to overcome fully activated I_KATP_ in *Kir6.2[K185Q,ΔN30]* β cells.

### LRRC8-mediated swell-secretion coupling is sufficient to induce insulin secretion in the presence of constitutive K_ATP_ activation.

**Figure 6:**
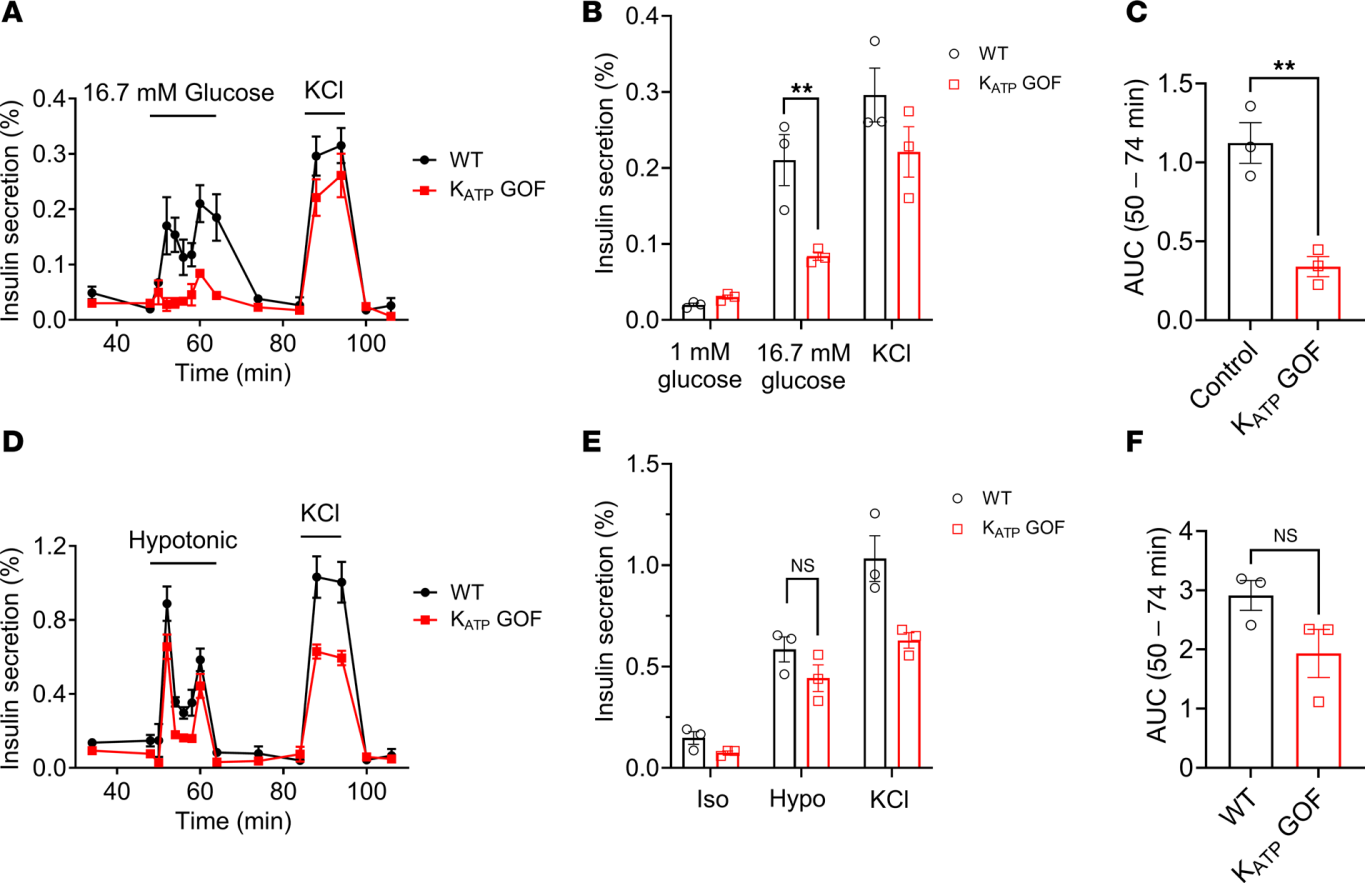
Hypotonic swelling induces insulin secretion in β cells independently of K_ATP_ channel closure. (**A**) Glucose-stimulated insulin secretion (16.7 mM) from islets isolated from WT (*n* = 3–4) and K_ATP_ GOF (*n* = 3–4) mice. Insulin secretion is represented as a percentage of the total insulin content. (**B**) Mean (%) insulin secretion from islets isolated from WT and K_ATP_ GOF mice in response to 1 mM glucose (48 min), 16.7 mM glucose (60 min), and 40 mM KCl (88 min). (**C**) Mean AUC for the percentage of insulin secretion during both first-phase and second-phase (50–74 min) responses to glucose (16.7 mM) stimulation. (**D**) Hypotonicity-induced insulin secretion (210 mOsm) from islets isolated from WT (*n* = 3–4) and K_ATP_ GOF (*n* = 3–4) mice. Insulin secretion is represented as a percentage of the total insulin content. (**E**) Mean (%) insulin secretion from islets isolated from WT and K_ATP_ GOF mice in response to isotonic solution (48 min), hypotonic solution (60 min), and 40 mM KCl (88 min). (**F**) Mean AUC for the percentage of insulin secretion during both first-phase and second-phase (50–74 min) responses to hypotonic (210 mOsm) solution. Data are represented as mean ± SEM. Statistical significance was evaluated using either a 2-tailed unpaired Student’s *t* test (for normally distributed data) or the Mann-Whitney test (for nonparametric comparisons) (**P < 0.01).

Next, we examined the ability of swelling alone to stimulate insulin secretion from both WT and K_ATP_ GOF islets. Similar to Ca^2+^ measurements shown in Figure 5, and consistent with previous reports (3, 38, 42), K_ATP_ GOF islets exhibited markedly suppressed GSIS in response to high (16.7 mM) glucose when compared with WT islets expressing wild-type K_ATP_ (Figure 6, A–C). However, K_ATP_ GOF islets remained responsive to hypotonic swelling to a level comparable to WT islets (Figure 6, D–F). In contrast to delayed hypotonically stimulated Ca^2+^ transients observed in isolated K_ATP_ GOF β cells (Figure 5I), K_ATP_ GOF islets did not exhibit a delay in insulin secretion. This apparent discrepancy between the delayed calcium response in dispersed K_ATP_ GOF β cells (Figure 5I) and the unchanged insulin secretion kinetics in intact islets (Figure 6D) likely reflect fundamental differences in the behavior of isolated β cells versus the intercellular dynamics within the syncytium of an intact islet. As demonstrated in studies of pancreatic islet function (43–46), intercellular synchronization via connexin-mediated gap junctions and paracrine signaling allows β cells to function as a coordinated network, reducing cell-to-cell variability and promoting integrated oscillatory calcium responses. Collectively, these data demonstrate that hypotonic swelling alone provides a sufficiently strong stimulus to drive insulin secretion independent of K_ATP_ channel closure and collectively supports the concept of swell-secretion coupling in pancreatic β cells.

## Discussion

**Figure 7:**
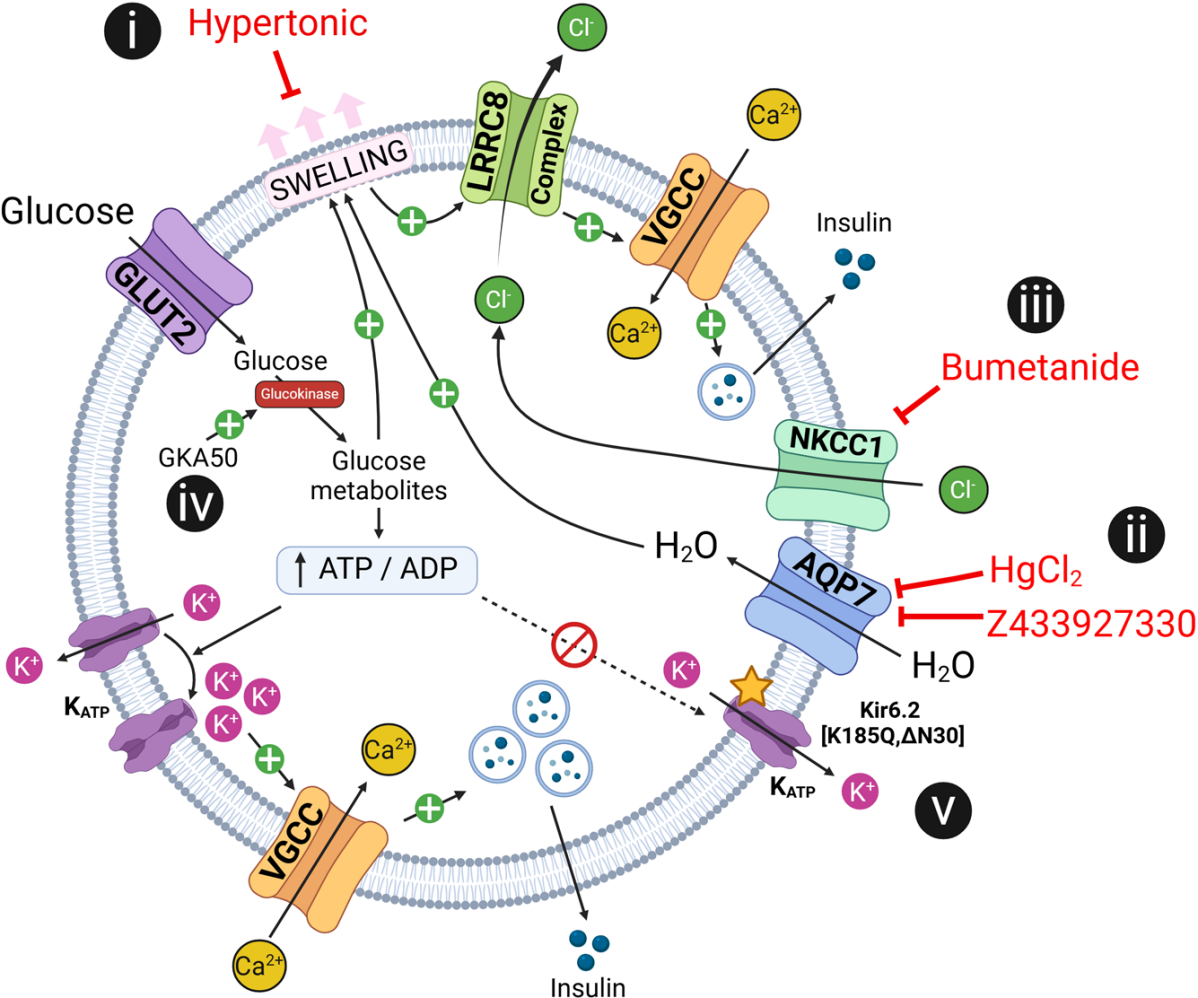
Testing a working model of LRRC8-mediated swell-secretion coupling counterbalancing KATP. Exposure of β cells to high glucose levels causes them to swell, activating LRRC8 channels. LRRC8 channel activation triggers a chloride ion current (I_Cl,SWELL_), leading to membrane depolarization. As the membrane depolarizes, VGCCs open, allowing Ca²^+^ ions to enter the cells. This influx of Ca²^+^ is essential for triggering insulin secretion, as the increased intracellular calcium stimulates the release of insulin-containing vesicles. (i) Suppression of osmotic swelling during glucose stimulation by application of hypertonic solution dose-dependently reduces insulin secretion. (ii) Blocking water influx via AQP7 channels suppresses β cell swelling and also markedly inhibits insulin secretion. (iii) Inhibiting the NKCC1 cotransporter with bumetanide to decrease [Cl^–^]_i_ in β cells reduces I_Cl,SWELL_-mediated depolarizing Cl^–^ efflux and impairs insulin secretion in response to both glucose stimulation and hypotonic swelling. (iv) Inducing β cell swelling by hyperactivating glucokinase with GKA50 is sufficient to trigger LRRC8A-dependent insulin secretion in islets. (v) Glucose-stimulated intracellular Ca^2+^ and insulin secretion are fully suppressed in β cells with constitutively active K_ATP_. However, hypotonic swelling is capable of overcoming K_ATP_ to stimulate both intracellular Ca^2+^ signaling and insulin. AQP7, aquaporin protein 7; GKA50, glucokinase activator 50; GLUT2, glucose transporter 2; K_ATP_, ATP-sensitive potassium channel; LRRC8, leucine-rich repeat containing 8 protein; NKCC1, sodium-potassium-chloride cotransporter 1; VGCC, voltage-gated calcium channel.

Previously, we reported that LRRC8 channel complexes contribute to cell-swelling mediated insulin secretion in vitro and in vivo (23). In this present study, we further tested this “swell-secretion” model by interrogating this proposed mechanism at several different levels (Figure 7). First, we suppressed osmotic swelling during glucose stimulation by purposefully increasing the osmolarity of the extracellular solution, and this was accompanied by a hypertonicity dose-dependent reduction in insulin secretion (Figure 7, i). Second, we suppressed swelling by blocking water influx via AQP7 channels, and this also markedly inhibited insulin secretion (Figure 7, ii), consistent with the observed reduction in GSIS in AQP7-KO mice (34). Third, inhibition of the NKCC1 cotransporter with bumetanide to decrease [Cl^–^]_i_ in β cells and reduce I_Cl,SWELL_-mediated depolarizing Cl^–^ efflux also impaired insulin secretion in response to both glucose-stimulation and hypotonic swelling (Figure 7, iii), consistent with prior intracellular Ca^2+^ recordings in glucose-stimulated MIN6 cells and isolated murine β cells (23), and as reported previously in islets (47). Fourth, inducing β cell swelling by hyperactivating glucokinase with GKA50 at substimulatory 4 mM glucose concentrations was sufficient to trigger insulin secretion in islets, and this insulin release was LRRC8A dependent (Figure 7, iv). Fifth, while glucose-stimulated intracellular Ca^2+^ and insulin secretion are fully suppressed in β cells with constitutively active K_ATP_, hypotonic swelling is capable of overcoming K_ATP_ to stimulate both intracellular Ca^2+^ signaling and insulin secretion (Figure 7, v). Collectively, these data further support a model of swell-secretion coupling, wherein mechanoresponsive LRRC8 anion channel complexes sense β cell swelling to potentiate β cell excitability and insulin secretion in concert with K_ATP_.

The application of hypertonic perfusate up to 380 mOsm to suppress β cell swelling was used as a mechanistic maneuver to assess the contribution of β cell swelling–stimulated I_Cl,SWELL_ to insulin secretion. However, clinically, serum osmolarity can rise up into the 400–450 mOsm range in diabetic ketoacidosis and hyperglycemic hyperosmolar states, which are both acute metabolic complications of diabetes mellitus that can occur in patients with both type 1 and 2 diabetes mellitus (48), as blood glucose values often exceed 1,000 mg/dL (48). In these clinical scenarios, LRRC8 channel activation will be markedly suppressed, and consequently, pancreatic β cell insulin secretion will be further impaired, which will only further exacerbate the hyperglycemic state. Indeed, fluid resuscitation and correction of the hyperosmolar state, as done clinically, are predicted to augment LRRC8 channel activity, improve insulin secretion, and contribute to improving the hyperglycemic state.

The ability of LRRC8 channels to depolarize the β cell depends on an elevated intracellular β cell Cl^–^ concentration, which is set by the NKCC1 cotransporter (49, 50). As demonstrated in this study with the NKCC1 inhibitor bumetanide, NKCC1 activity can itself regulate β cell insulin secretion and glucose homeostasis, and this is consistent with results from β cell–targeted NKCC1-KO mice, which also exhibit impaired GSIS (51). Indeed, it has been observed clinically that bumetanide and other loop diuretics acting on the NKCC1 cotransporter can cause hyperglycemia (52, 53), due to impaired insulin secretion (54). Based on the findings presented in the current study, it is plausible that these clinically observed side effects of bumetanide and other NKCC1 inhibitor loop diuretics to increase blood glucose may be due to reductions LRRC8-mediated I_Cl,SWELL_ currents in β cells — providing a mechanism for a longstanding clinical observation.

In pancreatic β cells, K_ATP_ channels, composed of the sulfonylurea receptor, SUR1, and Kir6.2 subunits, are regulated by intracellular nucleotides, inhibited by ATP, and activated by ADP (55). Their critical function in modulating insulin release from pancreatic islets has been well described (56–58). When blood glucose levels drop, β cell glucose concentrations decline, reducing the [ATP]/[ADP] ratio. This shift activates K_ATP_ channels, hyperpolarizing the β cell membrane, preventing activation of voltage-gated Ca²^+^ channels and associated Ca²^+^ influx, and suppressing insulin secretion. Our findings demonstrate that maximally activating LRRC8 channels with hypotonic swelling is sufficient to depolarize β cells and stimulate Ca^2+^ transients and insulin secretion in the presence of open K_ATP_ channels at low glucose levels as well as in the presence of constitutively activated K_ATP_ channels in the K_ATP_ GOF β cell by overcoming the hyperpolarizing currents flowing through K_ATP_ channels. In contrast, LRRC8 channel activation associated with glucose stimulation alone is incapable of activating K_ATP_ GOF β cells. These experiments highlight the counterbalancing relationship between LRRC8 and K_ATP_ channels.

While our study primarily examines the role of LRRC8 channels in β cell swelling–induced signaling, our findings suggest potential therapeutic strategies for insulin secretion disorders, such as neonatal diabetes caused by GOF K_ATP_ mutations. Neonatal diabetes associated with GOF K_ATP_ mutations results in constitutively open K_ATP_ channels, hyperpolarizing the β cell membrane and preventing insulin secretion (59, 60). Our data show that LRRC8 channel activation generates depolarizing current (due to Cl^–^ efflux) under hypotonic conditions, which counteracts K_ATP_-mediated hyperpolarization. Enhancing LRRC8A activity, therefore, represents a putative strategy to restore membrane depolarization and stimulate insulin release in β cells in the setting of K_ATP_ GOF mutations. Developing small molecules or peptides to enhance LRRC8 channel function could provide a novel approach to counterbalance the effects of hyperpolarization in insulin secretion disorders, particularly for patients with K_ATP_ GOF mutations. This pathway represents a promising target for therapeutic exploration and highlights the broader importance of LRRC8 channels in maintaining β cell function.

Although numerous mutations in K_ATP_ channel genes, such as those in ABCC8 and KCNJ11, are well documented in patients with insulin secretion disorders, the role of LRRC8 genetic variants in insulin secretion has not been thoroughly investigated (61–63). Genetic variants in LRRC8A and other LRRC8 subunits, such as LRRC8C have been identified in human populations and are primarily associated with immune deficiencies and developmental disorders (64, 65). For instance, mutations in LRRC8 have been linked to agammaglobulinemia, a condition characterized by an absence of immunoglobulins due to defective B cell development (64). Similarly, de novo variants in LRRC8C have been associated with multisystem disorders presenting with intellectual disability, microcephaly, and metaphyseal dysplasia (65). Despite the established roles of LRRC8 proteins in immune function and development, their involvement in pancreatic β cell function and insulin secretion has not been thoroughly investigated. Recent studies have demonstrated that LRRC8/VRAC anion channels enhance β cell glucose sensing and insulin secretion (23, 24). However, the specific impact of genetic variants in LRRC8 proteins on insulin secretion remains unexplored.

Aside from LRRC8 channel complexes described in this study, other mechanoresponsive or mechanosensitive ion channels have been proposed to be active in pancreatic β cells that may function in a similar manner and contribute to β cell depolarization via cation influx, rather than anion (Cl^–^) efflux (66). Piezo1, one of the most widely known and studied mechanosensitive ion channels, has been proposed to activate in response to mechanical forces associated with glucose metabolism and swelling within β cells and mediates inward cationic currents, which contribute to β cell depolarization and subsequent activation of VGCCs and insulin secretion (67). Similarly, TMEM150C (also known as tentonin 3 and TTN3), a nonselective cation channel with high calcium permeability (68), is also implicated in insulin secretion by promoting β cell depolarization via cationic influx (69). Finally, while several transient receptor potential (TRP) channels have been implicated in insulin secretion from pancreatic β cells (6), TRPV2 in particular has been reported to contribute to membrane depolarization and subsequent insulin secretion in murine β cells following mechanical activation by osmotic swelling (70). Overall, these studies collectively support a generalized model wherein mechanical force/swelling associated with glucose transport and metabolism in β cells can be sensed and transduced by certain ion channels, such as LRRC8 complexes, as a mechanism to promote insulin secretion from pancreatic islets.

## Methods

### Sex as a biological variable.

Both male and female mice were used in the studies, yielding similar findings across sexes. Consequently, sex was not considered a biological variable in this study. Experimental design, data collection, and analysis were conducted without sex-based stratification.

### Animals.

All experimental procedures involving mice were approved by the Institutional Animal Care and Use Committee of Washington University in St. Louis. *Lrrc8a*^fl/fl^ mice were generated as described previously (71). *Rosa26-Kir6.2[K185Q,ΔN30]* mice were generated as described previously (38). Mice were housed in a controlled environment with regulated temperature, humidity, and light cycles and were provided with free access to water and food. Both male and female mice (50% male, 50% female), aged 8–20 weeks, including *Lrrc8a*^fl/fl^ and *Rosa26-Kir6.2[K185Q,ΔN30]* mice, were used for in vitro experiments.

### Chemicals and reagents.

β Cell–targeted GCaMP6s (Ad5-RIP1-GCaMP6s, 4.9 × 10^10^ PFU/mL) and β cell–targeted GCaMP6s-2A-iCre (Ad5-RIP1-GCaMP6s-2A-iCre, 5.8 × 10^10^ PFU/mL) were obtained from Vector Biolabs. Bumetanide (B3023), glucokinase activator GKA50 (SML0849), mercury(II) chloride (215465), type V collagenase (C9263), and 1-(Ethoxycarbonylmethyl)-6-methoxyquinolinium bromide (MQAE; 46123) were purchased from Sigma-Aldrich. Z433927330 was obtained from MedChemExpress. Stock solutions of drugs were made in DMSO (D8418, Sigma-Aldrich) and diluted to desired concentrations immediately before use.

### Murine islet isolation.

*Lrrc8a*^ﬂ/ﬂ^ and *Rosa26-Kir6.2[K185Q,ΔN30]* mice, aged 8–20 weeks, were anesthetized by isoflurane, followed by cervical dislocation in accordance with the approved procedures. The pancreas was perfused via the common bile duct with 2–3 mL Hank’s Balanced Salt Solution containing 0.8 mg/mL type V collagenase. After perfusion, the pancreas was removed and digested at 37°C for 10 minutes. The islets were then isolated by gentle agitation, washed in RPMI medium with 2% FBS, and purified using Histopaque 1077 and 1119 gradients. The purified islets were placed in a 60 mm petri dish with culture medium for short-term (24 h) or long-term (up to 96 h) culture.

### Adenoviral transduction of murine islets.

Isolated murine islets from *Lrrc8a*^ﬂ/ﬂ^ and *Rosa26-Kir6.2[K185Q,ΔN30]* mice were cultured in RPMI medium supplemented with 2% FBS for 2 hours. On the same day, adenovirus encoding RIP-GCaMP6s or RIP-GCaMP6s-2A-iCre was introduced into the islet-containing medium at a final concentration of 5 × 10^7^ PFU/mL, followed by incubation for 12–16 hours. Islets were subsequently washed 3 times with PBS and then cultured in RPMI medium with 10% FBS for 1–5 days before further experimentation. The efficiency of transduction was evaluated using fluorescence microscopy.

### Insulin secretion assay.

Perifusion experiments to assess GSIS were conducted on isolated islets from *Lrrc8a*^ﬂ/ﬂ^ and *Rosa26-Kir6.2[K185Q,ΔN30]* mice using a PERI4-02 from Biorep Technologies. For each experiment, around 30–50 freshly isolated islets (all from the same isolation batch) were handpicked to match the size of islets across the samples and loaded into the polycarbonate perifusion chamber between two layers of polyacrylamide-microbead slurry (Bio-Gel P-4, Bio-Rad Laboratories). For glucose-induced secretion, perfusing buffer contained 120 mM NaCl, 24 mM NaHCO_3_, 4.8 mM KCl, 2.5 mM CaCl_2_, 1.2 mM MgSO_4_, 10 mM HEPES, 1 or 4 mM glucose, 29 or 26 mM mannitol, 0.1% w/v bovine serum albumin, pH 7.4, with NaOH (300 mOsm/kg); for hypotonicity-induced secretion, perfusing buffer contained 90 mM NaCl, 5 mM NaHCO_3_, 4.8 mM KCl, 1.2 mM KH_2_PO_4_, 2.5 mM CaCl_2_, 2.4 mM MgSO_4_, 10 mM HEPES, 0.1% w/v bovine serum albumin, pH 7.4, with NaOH (210 mOsm/kg). Perfusing buffers kept at 37°C were circulated at 120 μL/min. After 48 minutes of washing with 1 or 4 mM glucose solution or isotonic solution for stabilization, islets were stimulated with the following sequence: 16 minutes of 16.7 mM glucose or hypotonic solution, 20 minutes of basal glucose (1 or 4 mM, depending on the specific experiment) or isotonic solution, 10 minutes of 30 mM KCl, and 12 minutes of basal glucose (1 or 4 mM, depending on the specific experiment) or isotonic solution. Osmolarity was matched by adjusting the mannitol concentration when preparing solutions containing various concentrations of glucose or isotonic solution. The osmolarity of hypertonic solutions (330, 360, and 380 mOsm/kg) were also adjusted by using mannitol. Serial samples were collected either every 1–2 minutes into 96-well plates and kept at 4°C. Insulin concentrations were determined with commercially available ELISA kits (10-1247-01, Mercodia Mouse Insulin ELISA). At the completion of the experiments, islets were lysed by the addition of ethanol buffer, and the amount of insulin was detected by ELISA. Data were obtained from 3 independent experiments and normalized to total insulin contents.

### Cell volume measurements.

To investigate β cell swelling induced by glucose or glucokinase activators, WT and LRRC8A-KO dissociated primary β cells were imaged using bright-field microscopy. Imaging was conducted at 37°C using an Olympus IX73 microscope equipped with a ×40/0.60 NA objective lens and a CMOS CCD camera for data acquisition. Images were captured at 10-second intervals using MetaMorph software (Molecular Devices). β Cells were initially perfused with a basal Krebs-Ringer Bicarbonate HEPES (KRBH) solution containing 129 mM NaCl, 5 mM NaHCO_3_, 4.8 mM KCl, 1.2 mM KH_2_PO_4_, 2.5 mM CaCl_2_, 2.4 mM MgSO_4_, 10 mM HEPES, 4 mM glucose, 26 mM mannitol, pH 7.4, with NaOH (300 mOsm/kg). Following this, the glucokinase activator GKA50 was introduced into the perfusion system. Quantification of the β cell cross-sectional area was performed as described previously (23) and was obtained from bright-field images via ImageJ (NIH) using an automated tool and expressed as a total cross-sectional area (*A*) over an initial area (*A_o_*).

### Calcium imaging.

Dispersed islets, isolated from *Lrrc8a*^ﬂ/ﬂ^ and *Rosa26-Kir6.2[K185Q,ΔN30]* mice and transduced with GCaMP6s and GCaMP6s-2A-iCre, were incubated at 37°C for 30 minutes in basal isotonic KRBH buffer consisting of 90 mM NaCl, 5 mM NaHCO_3_, 4.8 mM KCl, 1.2 mM KH_2_PO_4_, 2.5 mM CaCl_2_, 2.4 mM MgSO_4_, 10 mM HEPES, 90 mM mannitol, pH 7.4, with NaOH (300 mOsm/kg) after the removal of culture medium. For calcium imaging experiments with hypotonic swelling, hypotonic KRBH buffer consisted of the following: 90 mM NaCl, 5 mM NaHCO_3_, 4.8 mM KCl, 1.2 mM KH_2_PO_4_, 2.5 mM CaCl_2_, 2.4 mM MgSO_4_, 10 mM HEPES, pH 7.4, with NaOH (210 mOsm/kg). For glucose-stimulated intracellular calcium signaling, primary β cells were perfused with a basal KRBH solution containing 129 mM NaCl, 5 mM NaHCO_3_, 4.8 mM KCl, 1.2 mM KH_2_PO_4_, 2.5 mM CaCl_2_, 2.4 mM MgSO_4_, 10 mM HEPES, 1 mM glucose, 29 mM mannitol, pH 7.4, with NaOH (300 mOsm/kg), and the glucose concentration was increased from 1 to 16.7 mM for stimulation. Osmolarity was matched by adjusting with mannitol. All imaging was performed at 35–37°C. For murine β cells, dissociated β cells were imaged with a ×20/0.75 NA objective (Olympus) on an Olympus IX73 microscope and were imaged every 10 seconds via 485 nm excitation and 520 nm emission filters; relative changes in calcium concentration were expressed as *F*/*F_o_*.

### β Cell intracellular chloride imaging.

Intracellular chloride levels in β cells were measured using the Cl^–^-sensitive fluorescent dye MQAE. A 500 mM stock solution of MQAE was prepared in DMSO and stored at −20°C. Dissociated primary β cells were incubated with 5 mM MQAE for 60 minutes at 37°C, a widely used protocol for intracellular chloride measurement across various cell types (72–74). Following incubation, MQAE-loaded cells were plated onto Matrigel-coated coverslips. Before imaging, cells were transferred to a perfusion chamber and bathed in an extracellular low Cl^–^ solution (24 mM Cl^–^) containing 120 mM L-aspartic acid, 20 mM CsCl, 1 mM MgCl_2_, 1 mM CaCl_2_, 5 mM HEPES, 120 mM CsOH, and 2.8 mM glucose, adjusted to pH 7.4 with CsOH (300 mOsm/kg). The total cellular fluorescence intensity (*F*) was used to determine the intracellular chloride concentration ([Cl^–^]ᵢ) between treated and untreated β cells, and relative changes in [Cl^–^]ᵢ were determined by normalizing total cellular fluorescence intensity to the initial cellular fluorescence intensity (*F_o_*) to report *F*/*F_o_*. β Cells were incubated in 20 M bumetanide for 1 hour before MQAE imaging. In another set of experiments, cells were transferred from media (140 mM Cl^–^) to a low-Cl^–^ solution (24 mM Cl^–^) and then perfused with a high-Cl^–^ solution (135 mM Cl^–^) containing 125 mM NaCl, 5 mM NaHCO_3_, 4.8 mM KCl, 1.2 mM KH_2_PO_4_, 2.5 mM CaCl_2_, 2.4 mM MgSO_4_, 10 mM HEPES, and 2.8 mM glucose (pH 7.4, adjusted with NaOH). MQAE fluorescence imaging was performed using an Olympus IX73 microscope equipped with a ×20/0.75 NA objective lens and fura-2 cube; images were captured using 340 nm excitation and 508 nm emission filters and a Lambda DG-4 illumination system and imaged with a Hamamatsu Orca Flash 2.0 CMOS CCD camera. Images were captured using MetaMorph software (Molecular Devices).

### Statistics.

Data are presented as mean ± SEM. The Shapiro-Wilk test was performed to assess the normality of each dataset. For normally distributed data, statistical significance was determined using a 2-tailed unpaired Student’s *t* test (when comparing only 2 groups) or 1-way ANOVA (when comparing 3 or more groups). For nonnormally distributed data, the Mann-Whitney test was applied. A probability of *P* < 0.05 was considered to be statistically significant.

### Study approval.

All experimental procedures involving mice were approved by the Institutional Animal Care and Use Committee of Washington University in St. Louis.

### Data availability.

All data used in the paper are available. Values for all data points in graphs are reported in the Supporting Data Values file.

## Author contributions

CK, TMAEA, RC, and RS designed research studies. CK, TMAEA, LX, SP, and RS conducted experiments, analyzed data, and prepared figures. CK, TMAEA, JDT, and RS wrote the manuscript. CK, TMAEA, JDT, RC, MSR, and RS revised the manuscript. All authors reviewed and approved the manuscript.

## Supplementary Material

Supplemental data

Supporting data values

## Version 1 04/29/2025

In-Press Preview

## Version 2 06/09/2025

Electronic publication
