# Supplementary material for: LRRC8 channel complexes counterbalance KATP channels to mediate swell-secretion coupling in mouse pancreatic β cells
Source: JCI Insight. 2025 Apr 29;10(11):e188020. doi: 10.1172/jci.insight.188020 (PMC12220942; doi:10.1172/jci.insight.188020)
Supplement: Supplemental data [file jciinsight-10-188020-s219.pdf]

LRRC8 channel complexes counterbalance K_{ATP} channels to mediate swell-secretion coupling in mouse pancreatic β-cells

Tarek Mohamed Abd El-Aziz[1,2,*], Chen Kang[1,*], Litao Xie[1], John D. Tranter[1], Sumit Patel[3], Rahul Chadda[1], Maria S. Remedi[3], Rajan Sah[1,4]

[1]Department of Internal Medicine, Cardiovascular Division, Washington University School of Medicine, St. Louis, Missouri, USA

[2]Zoology Department, Faculty of Science, Minia University, El-Minia, Egypt

[3]Department of Medicine, Division of Endocrinology, Metabolism and Lipid Research, Washington University School of Medicine, St. Louis, MO, USA

[4]St. Louis VA Medical Center, St. Louis, Missouri, USA

*These authors contributed equally

Address correspondence to: Rajan Sah, BJC Institute of Health, 425 S Euclid Ave, St. Louis, MO 63110, USA. Phone: +1-314-273-7748; Email: rajan.sah@wustl.edu

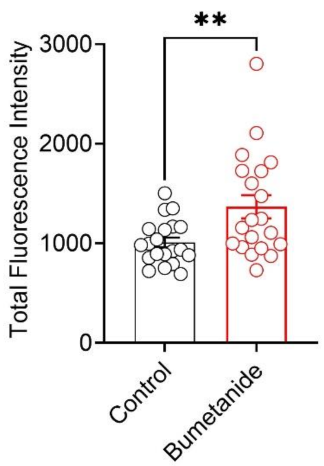

Supplementary Figure 1. Bumetanide treatment reduces intracellular Cl^- concentration in β-cells. Total MQAE fluorescence intensity in β-cells under control conditions (n = 20) and after treatment with 20 μM bumetanide (n = 20). Data are presented as mean ± S.E.M. Statistical significance for all data was determined using two-tailed unpaired Student's t-test (**P < 0.01).

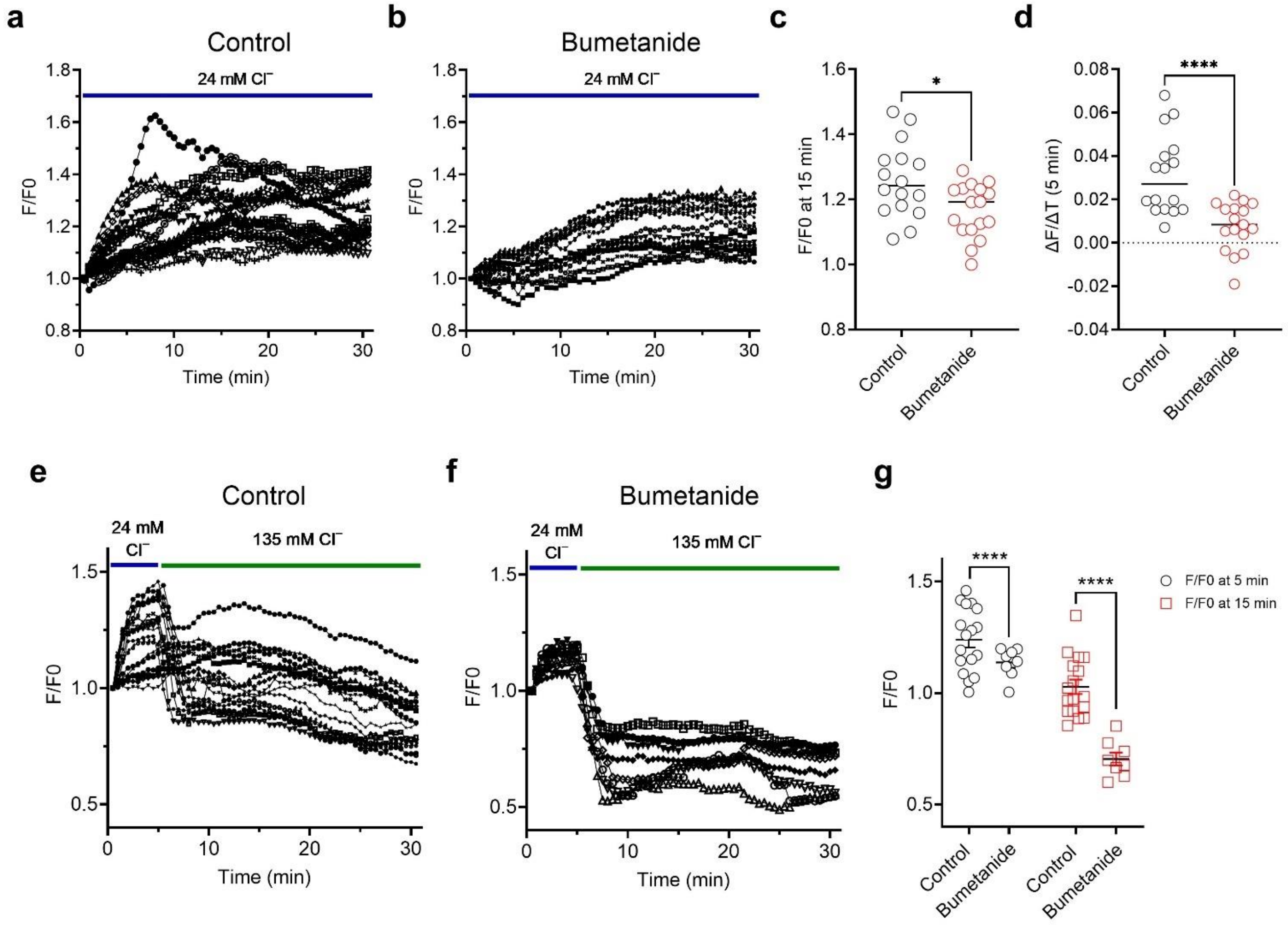

Supplementary Figure 2. Bumetanide pre-treatment lowers β-cell intracellular Cl^- as compared to control thereby reducing Cl^- efflux and increasing Cl^- influx. a,b Normalized MQAE fluorescence in control (n = 16) and bumetanide-treated β-cells (n = 17) during perfusion with a low-Cl^- solution (24 mM). **c** Mean normalized MQAE fluorescence in control and bumetanide-treated β-cells after 15 minutes of perfusion with a low-Cl^- solution (24 mM). **d** Rate of change of MQAE fluorescence (ΔF/ΔT) over 5 minutes in control and bumetanide-treated β-cells upon perfusion with a low-Cl^- (24 mM) solution. **e,f** Normalized fluorescence intensity during the transition from media to a low-Cl^- solution (24 mM), and then to high-Cl^- solution (135 mM) in control (n = 17) versus bumetanide-treated β-cells (n = 8). **g** Mean normalized MQAE fluorescence in control and bumetanide-treated β-cells after 5 minutes of perfusion with a low-Cl^- solution (24 mM) and 15 minutes of perfusion with a high-Cl^- solution (135 mM). Statistical significance for all data was determined using two-tailed unpaired Student's t-test (*$P < 0.05$; ****$P < 0.0001$).
